# The Cell-Free Integration of a Polytopic Mitochondrial Membrane Protein into Liposomes Occurs Cotranslationally and in a Lipid-Dependent Manner

**DOI:** 10.1371/journal.pone.0046332

**Published:** 2012-09-25

**Authors:** Ashley R. Long, Catherine C. O'Brien, Nathan N. Alder

**Affiliations:** Department of Molecular and Cell Biology, University of Connecticut, Storrs, Connecticut, United States of America; Auburn University, United States of America

## Abstract

The ADP/ATP Carrier (AAC) is the most abundant transporter of the mitochondrial inner membrane. The central role that this transporter plays in cellular energy production highlights the importance of understanding its structure, function, and the basis of its pathologies. As a means of preparing proteoliposomes for the study of membrane proteins, several groups have explored the use of cell-free translation systems to facilitate membrane protein integration directly into preformed unilamellar vesicles without the use of surfactants. Using AAC as a model, we report for the first time the detergent-free reconstitution of a mitochondrial inner membrane protein into liposomes using a wheat germ-based *in vitro* translation system. Using a host of independent approaches, we demonstrate the efficient integration of AAC into vesicles with an inner membrane-mimetic lipid composition and, more importantly, that the integrated AAC is functionally active in transport. By adding liposomes at different stages of the translation reaction, we show that this direct integration is obligatorily cotranslational, and by synthesizing stable ribosome-bound nascent chain intermediates, we show that the nascent AAC polypeptide interacts with lipid vesicles while ribosome-bound. Finally, we show that the presence of the phospholipid cardiolipin in the liposomes specifically enhances AAC translation rate as well as the efficiency of vesicle association and integration. In light of these results, the possible mechanisms of liposome-assisted membrane protein integration during cell-free translation are discussed with respect to the mode of integration and the role of specific lipids.

## Introduction

Membrane proteins constitute roughly one third of all gene products in any given organism, and over half of all current pharmaceutical targets [1], [2]. However, solution-based biochemical and biophysical studies of membrane proteins are technically challenging because their hydrophobicity causes them to form insoluble aggregates in aqueous systems. *In vivo* expression of such proteins, while successful in certain cases, can be hampered due to cell toxicity, misfolding, and aggregation [1], [3]. For these reasons, cell-free synthesis of membrane proteins is becoming recognized as a powerful technique for studying membrane proteins within model membrane systems [1], [2], [4]. By one strategy, cell-free protein synthesis is conducted in the presence of detergents to maintain the solubility of the translation product before reconstitution into liposomes [5], [6]. However, this approach can suffer drawbacks because even mild detergents with a low critical micelle concentration (cmc) can be inhibitory to the translation apparatus and can be difficult to remove [1], [2]. Recently a number of independent groups have reported that membrane proteins, even topologically complex ones, can integrate into pre-formed unilamellar liposomes during cell-free translation reactions and fold into a functional state without the inclusion of detergents or a dedicated complex that mediates integration [1], [2], [7]–[9]. Examples include proteins translated in cell-free systems based on *E.coli* lysates (i.e. bacteriorhodopsin [7], connexin-43 [8] and the F_o_/F_1_ ATP synthase [10]) as well as wheat germ lysates (i.e. stearoyl-CoA desaturase [9] and sphingolipid synthase [11]). By this experimental approach, some polypeptides require a specific lipid composition for unassisted integration. In the case of bacteriorhodopsin, for example, it was shown that the component lipids must have acyl chain lengths and/or degrees of unsaturation that maintain the bilayer above the phase transition temperature and excess lipid with inverted hexagonal (H_II_) phase propensity may block insertion [7]. In other cases, it has been suggested that liposomes must be present in the biosynthetic reaction for insertion to occur, supporting a cotranslational mode of integration [8]. In this study we have investigated the cell-free spontaneous integration of the mitochondrial ADP/ATP Carrier (AAC).

AAC is the most abundant transporter of the mitochondrial inner membrane (IM) [12]. As a member of the mitochondrial carrier family, it contains three modular repeats of two transmembrane segments (TMSs), each connected by matrix-facing loops with small helical elements [12]–[14]. As part of the oxidative phosphorylation system, this carrier mediates adenine nucleotide transport through the 1∶1 electrogenic exchange of ADP and ATP across the IM [15]. Hence AAC plays a central role in cellular energy metabolism. During transport AAC is believed to cycle between two extreme conformers [12], [15]. In the c-state the channel lumen is exposed to the cytosol and in the m-state the lumen is exposed to the matrix [12], [15]. These conformations can be stabilized by the specific inhibitors carboxyatractyloside (CAT) and bongkrekic acid, respectively [12], [15].

The biogenesis route of AAC within the cell is well established. AAC is encoded in nuclear DNA, translated on cytosolic ribosomes, and post-translationally integrated into the mitochondrial IM via the TIM22 (Translocase of the Inner Mitochondrial Membrane) pathway (or Carrier pathway) in a manner dependent upon the membrane potential of the IM [16]–[18]. As with many other mitochondrial membrane proteins, the biogenesis of AAC requires the presence of the dimeric phospholipid cardiolipin (CL), which is present in the IM at a concentration of 15–20% total phospholipid [19]–[25]. Following integration, AAC interacts with respiratory complexes as well as other members of the mitochondrial carrier family, such as the phosphate carrier, the dicarboxylate transporter and the GDP/GTP transporter in a CL-dependent fashion [22]. This is consistent with the observed role of CL in maintaining the association of IM respiratory complexes as supercomplexes [21], [22]. The activity of AAC is directly dependent on the presence of CL [19], [20]. A specific role for CL in AAC function is also supported by published x-ray structures, which reveal specific interactions of the CL headgroups and acyl chains at the matrix-facing regions of AAC [14].

The ability to reconstitute multi-spanning mitochondrial membrane proteins into liposomes by a detergent-free process would represent a significant technical advance for structural and functional studies. In this report, we show that AAC translated in a wheat germ lysate-based system integrates into small unilamellar vesicles (SUVs) in the absence of translocons and that it is functional as an adenine nucleotide transporter. This system does not recapitulate the *in vivo* import or folding of AAC, but rather serves as a suitable mimic for reconstitution into an isolated lipid environment. Within this system, we thoroughly characterize the role of CL with respect to both the rate of protein synthesis and the efficiency of integration. Finally, we provide strong experimental evidence that spontaneous membrane protein integration by this process is obligatorily cotranslational.

## Results

### I. In vitro translated AAC integrates directly into liposomes as functionally active carriers

#### A. The experimental system

When synthesized in a cell-free system, hydrophobic proteins typically result in the formation of insoluble precipitates. Detergents can, in some cases, be added to cell-free translation reactions to maintain membrane proteins soluble in micelles [26]–[28]. However, we found that nonionic detergents like *n*-Dodecyl-β-D-maltoside, even at low concentrations (0.05% [w/v]), were inhibitory to our wheat germ translational apparatus and resulted in lowered yields. We therefore adopted the recently-described procedure for spontaneous insertion of *in vitro* translated membrane proteins into liposomes [7]–[11], [29] to test whether AAC would properly integrate into SUVs included in our wheat germ lysate system.

**Figure 1:**
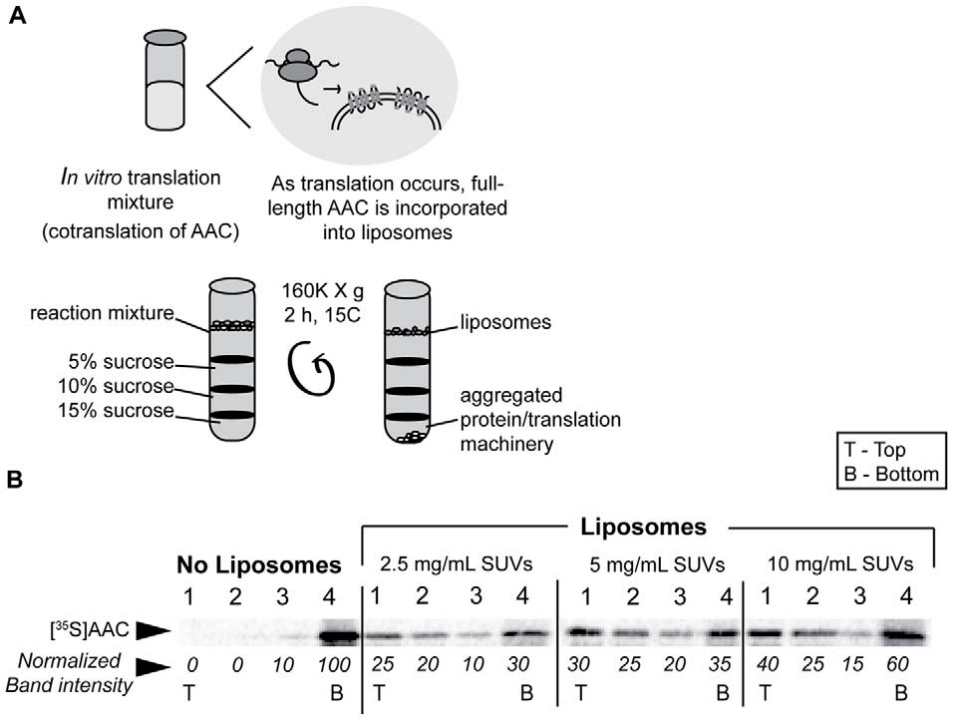
Cell-free translation and purification of AAC-containing proteoliposomes. A) Schematic illustration of the experimental process. [^35^S]AAC is translated in a wheat germ-based system in the presence of SUVs and the sample is subjected to SGU. Buoyant proteoliposomes remain at the top of the gradient, whereas aggregated protein and ribosomes from the translation reaction pellet. B) The extent of AAC integration depends on liposome concentration. [^35^S]AAC was translated in the presence of variable SUV concentrations as indicated and samples from each fraction were resolved by SDS-PAGE. Fractions 1–4 indicate fractions collected from top to bottom of the gradient. Normalized band intensities are shown below each lane of the gel.

In our experimental approach, unilamellar liposomes of a defined lipid content and size were added directly to the translation reaction and the resulting proteoliposomes were subsequently purified by discontinuous sucrose gradient ultracentrifugation (SGU) (Fig. 1A). These liposomes contained a biomimetic blend of 1-palmitoyl-2-oleoyl-*sn*-glycero-3-phosphocholine (POPC), 1-palmitoyl-2-oleoyl-*sn*-glycero-3-phosphoethanolamine (POPE), 1′,3′-bis[1,2-dioleoyl-*sn*-glycero-3-phospho]-*sn*-glycerol (CL), 1-palmitoyl-2-oleoyl-*sn*-glycero-3-phosphoserine (POPS), and 1-palmitoyl-2-oleoyl-*sn*-glycero-3-phosphate (POPA), in molar ratios of 54∶24∶16∶4∶2, respectively. To confirm that our translated AAC was integrated into liposomes, SGU was used to separate proteoliposomes from cell-free extract and any aggregated proteins (Fig. 1A). This method allows for the isolation of proteoliposomes from the translation components and unincorporated protein; proteoliposomes will float to the top of the sucrose gradient, whereas excess translation machinery will pellet [8]. By this analysis, [^35^S]AAC translated in the absence of liposomes pelleted as water-insoluble aggregates (Fig. 1B, “no liposomes”). In contrast, a fraction of [^35^S]AAC translated in the presence of liposomes floated with the vesicles, and the percentage of total AAC in the soluble liposome fraction increased with increasing liposome concentration (Fig. 1B, “liposomes”). We conclude that [^35^S]AAC associated strongly enough with SUVs that some fraction was recovered as floated proteoliposomes.

#### B. Assaying the proper integration of AAC

**Figure 2:**
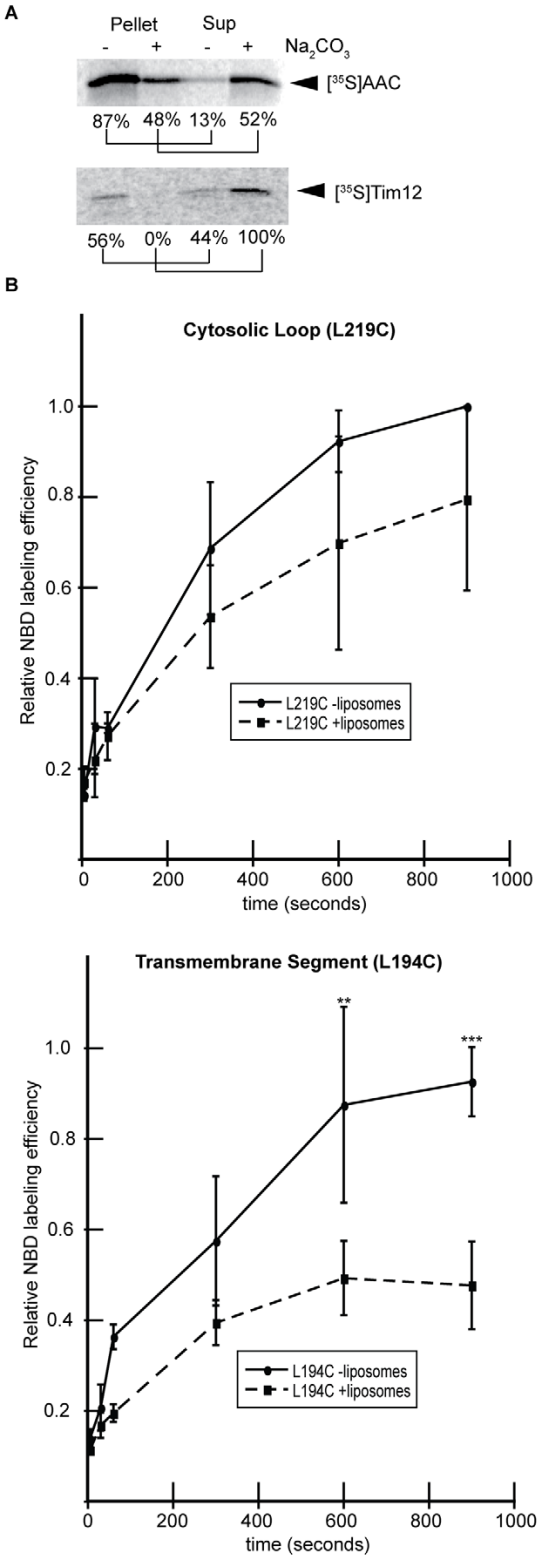
Assays for AAC integration into liposomes. A) Proteoliposomes containing the indicated radiolabeled proteins were incubated in the presence and absence of 100 mM Na_2_CO_3_ (pH 11.5). Following carbonate extraction, pellets and TCA-precipitated supernatants were resolved by SDS-PAGE. The percentage of protein per sample has been indicated. B) Site-specific labeling of AAC monocysteine variants with thiol-reactive NBD. AAC constructs with single cysteine residues on either the second cytosolic loop (AAC L219C, top panel) or transmembrane segment four (AAC L194C, bottom panel) were translated in the presence or absence of liposomes as indicated and subjected to a labeling reaction time course with IANBD. After quenching the reaction, samples were resolved by SDS-PAGE and the relative extent of labeling was quantified by in-gel fluorometry. The mean labeling efficiencies of four independent measurements is shown with standard deviations. Where indicated, the extent of labeling between samples with and without liposomes was significantly different (p<0.01 [**] and p<0.001 [***]) based on two-tailed t-tests.

We conducted a series of tests to address whether [^35^S]AAC translated in the presence of liposomes was properly integrated with transmembrane topology or nonspecifically bound to the vesicles. We first subjected our purified samples to alkaline extraction (pH 11.5), which will release peripheral membrane proteins from the bilayer but leave integral membrane proteins associated with the membrane [5], [30]. Following carbonate extraction, approximately 50% of [^35^S]AAC translated in the presence of SUVs remained stably associated with the vesicles (Fig. 2A, upper gel, compare pellet fractions with and without carbonate treatment). As a control, we translated the peripheral protein [^35^S]Tim12 in the presence of liposomes and found that all of the bilayer-associated protein was removed by carbonate (Fig. 2A, bottom gel, compare pellet fractions with and without carbonate treatment), confirming the efficiency of this procedure. These results indicate that a significant fraction of AAC spontaneously integrated into liposomes is carbonate resistant.

As an independent assay for the proper integration of our samples, we measured the solvent accessibility of single cysteine residues within AAC to the thiol-reactive derivative of the fluorescent probe 7-nitrobenz-2-oxa-1,3-diazolyl (IANBD). This membrane-impermeable reagent will react with cysteine residues in a polar environment, but not with those buried within a lipid bilayer [31]. Thus, we translated AAC variants containing a single cysteine on an intermembrane space-facing loop (AAC L219C) or in a transmembrane segment (AAC L194C) in the presence or absence of liposomes, subjected the samples to IANBD labeling, and quantified relative labeling efficiencies by in-gel fluorometry. The cysteine side chain in the loop region was labeled to a similar extent in the presence and absence of liposomes (Fig. 2B top panel, compare solid and dashed lines). In contrast, whereas the cysteine within the TMS was highly accessible to the labeling reagent in the absence of liposomes (Fig. 2B bottom panel, solid line), when translated in the presence of liposomes, its labeling decreased dramatically (Fig. 2B bottom panel, dashed line), consistent with its presence in a lipid bilayer. The [^35^S]AAC L194C polypeptides that were labeled in the presence of liposomes likely represent the fraction that was not fully integrated and/or misfolded (Fig. 2A). Taken together, the data from Fig. 2 indicate that a significant fraction of AAC translated in the presence of SUVs (roughly half, based on our results) is properly integrated into the membrane bilayer.

#### C. AAC is functionally active in cell-free generated proteoliposomes

**Figure 3:**
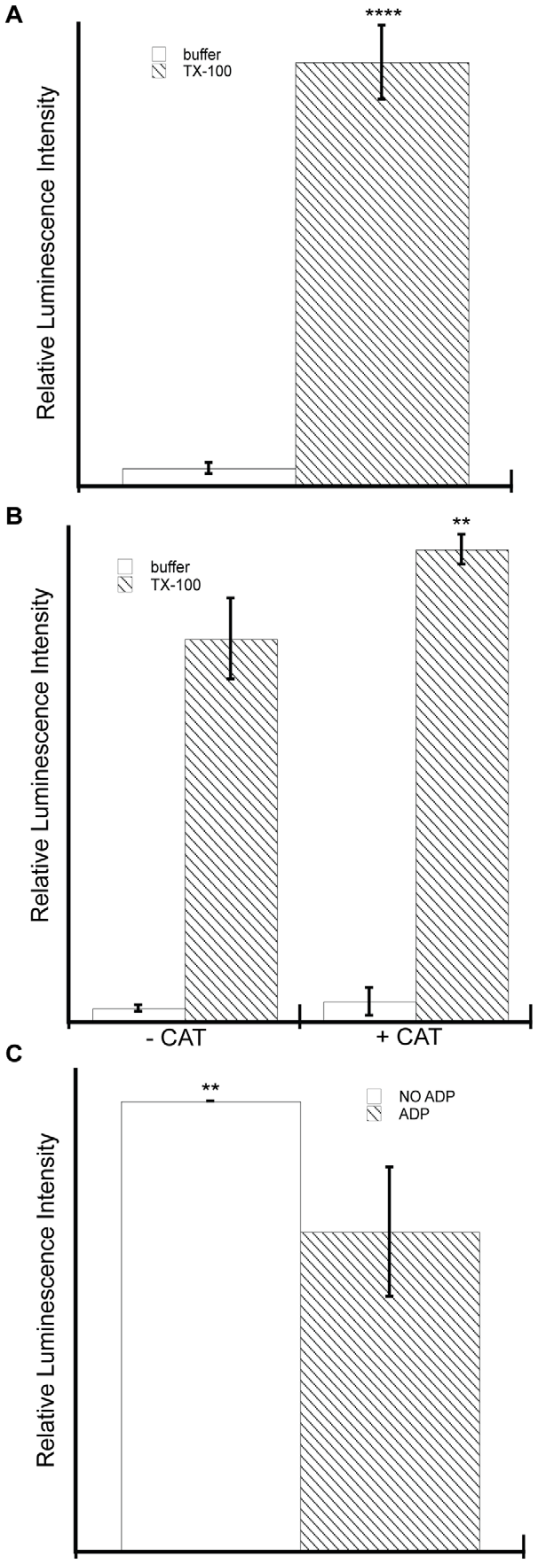
*In vitro* translated AAC in proteoliposomes is transport competent. Liposomes (A) or AAC-containing proteoliposomes (B, C) pre-loaded with ATP were incubated in the translation reaction to allow transport to occur. Vesicles were then purified by size-exclusion chromatography to remove unencapsulated nucleotide and assayed for total ATP content by luminescence immediately after disruption with detergent (0.2% triton X-100). A) Luminescence measurements of ATP-loaded liposomes pre-incubated with buffer only (white bar) or with detergent (hatched bar) registered a higher luminescence signal (p<0.0001 [****], two-tailed t-test) following disruption of the vesicles to release ATP. B) AAC-proteoliposomes with encapsulated ATP were prepared in the presence or absence of CAT as indicated and subjected to pretreatment with buffer only (white bars) or detergent (hatched bars) prior to luminescence measurements. The internal ATP content of proteoliposomes without CAT was significantly lower than samples prepared with CAT (p<0.01 [**], two-tailed t-test). The presence of CAT itself had no effect on the luminescence readings. C) AAC-proteoliposomes were prepared as in panel B, then incubated with buffer only (white bar) or 0.2 mM ADP (hatched bar), purified by a second round of gel filtration, and assayed for total luminal ATP content after detergent disruption. The internal ATP content of proteoliposomes subjected to ADP incubation was significantly lower than samples incubated with buffer only (p<0.01 [**], two-tailed t-test). All relative luminescence intensity measurements are means from a minimum of three independent experiments with standard deviations.

To assay the activity of our reconstituted AAC, we adapted the luciferin-luciferase assay [32] to test the ability of the carrier to specifically transport ATP out of lipid vesicles. In our experimental strategy, AAC translation reactions were supplemented with liposomes that were pre-loaded with ATP and the resulting proteoliposomes were incubated in the presence of the translation reaction to allow ADP/ATP transport to occur. Following gel filtration chromatography to remove excess nucleotide, the transport competence of AAC was assayed as the amount of ATP remaining in the liposomes by disrupting the vesicles with detergent and measuring the total ATP in the sample by luminescence. To illustrate the principle behind this strategy, ATP-loaded, column purified liposomes (not containing AAC) pre-incubated with buffer only registered a very low luminescence signal, indicating that there was little free ATP accessible to the luminescence reagent (Fig. 3A, white bar). However, when parallel samples of ATP-loaded vesicles were pre-incubated with triton X-100 just prior to measurement, the luminescence signal increased dramatically, demonstrating the high ATP content of the vesicle lumens (Fig. 3A, hatched bar).

We then applied this same analysis to AAC-containing proteoliposomes that were prepared in the presence and absence of the specific AAC inhibitor CAT (Fig. 3B). As expected, detergent treatment resulted in an increased luminescence signal showing that the purified proteoliposomes contained ATP (Fig. 3B, compare white and hatched bars for both “−CAT” and “+CAT” samples). However, AAC proteoliposomes prepared in the presence of inhibitor retained a significantly higher amount of ATP than those prepared without inhibitor (Fig. 3B, compare hatched bars of “+CAT” and “−CAT” samples). These results show that during the incubation process, ATP was specifically transported out of the proteoliposomes containing non-inhibited AAC. The decrease in luminal ATP in this assay was likely from AAC-mediated antiport of [ATP]^in^ (at high concentration) and [ADP]^out^ (present in the translation reaction), although an AAC-mediated uniport of ATP out of the liposomes (half-reaction) may have occurred as well [33].

As an independent means of measuring ADP/ATP antiporter activity of our reconstituted AAC, we subjected column-purified, ATP-loaded proteoliposomes to an incubation step with 200 µM ADP (or buffer only) for 30 min, re-purified them by gel filtration to remove excess nucleotide, and assayed for encapsulated ATP as above. Proteoliposomes incubated with ADP contained significantly lower ATP concentrations than those incubated with buffer alone (Fig. 3C, compare white and hatched bars), confirming that the presence of ADP stimulated the efflux of ATP from the proteoliposomes. Although these results do not provide an exact measure of transporter specific activity, they do confirm that our AAC-containing proteoliposomes support adenine nucleotide transport and are therefore in a functionally active state.

### II. The synthesis and integration of AAC is enhanced in the presence of CL-containing liposomes

**Figure 4:**
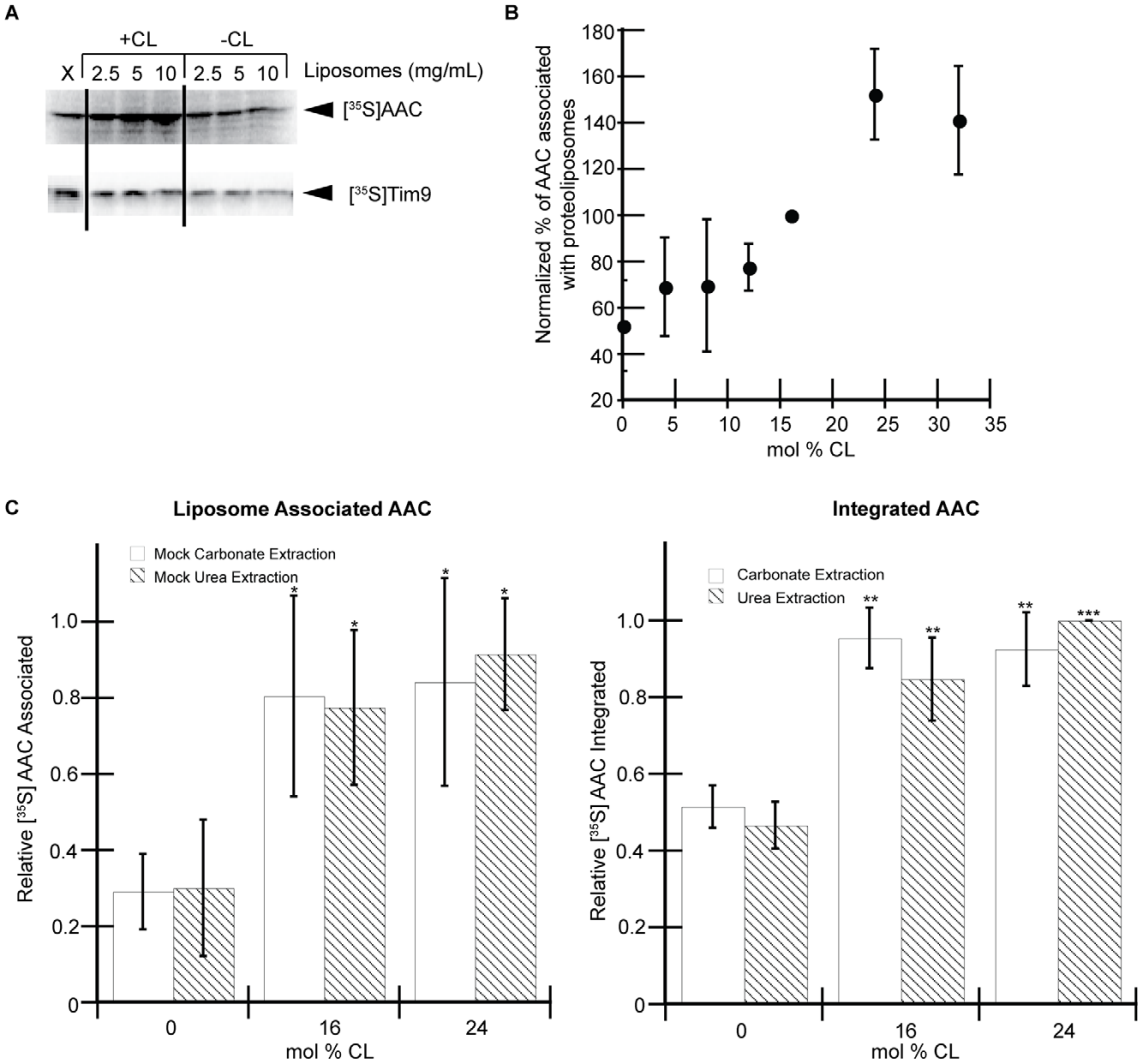
The presence of CL in liposomes enhances AAC synthesis and membrane integration. A) Liposome dependence of total protein synthesis. Cell-free translation reactions were programmed with mRNA encoding AAC or Tim9 in the absence of liposomes (“X”) or in the presence of liposomes at the final lipid concentration shown. B) Effect of CL concentration on AAC-liposome association. AAC translation reactions were conducted in the presence of liposomes containing variable mol% CL as indicated and purified by SGU. The relative amounts of co-isolated [^35^S]AAC are shown as mean values from a minimum of three independent experiments with standard deviations. C) Effect of CL concentration on AAC integration. [^35^S]AAC-containing proteoliposomes with variable amounts of CL were prepared and purified by SGU as in panel B. A subset of the samples were subjected to mock (buffer only) treatment (left panel) and the remaining samples were subjected to carbonate or urea extraction (right panel). Values shown are average band intensities normalized with respect to the highest value for liposome associated (left) or fully integrated (right) sample sets from three independent experiments. The relative amount of [^35^S]AAC in mock-treated samples (left), with 16 or 24 mol% CL, is significantly higher (p<0.05 [*], two tailed t-test) than with 0 mol% CL. In carbonate- and urea- treated samples (right) the difference in the relative amount of [^35^S]AAC present in the 16 and 24 mol% CL samples (compared to 0%) is even greater than in the untreated samples (p<0.01 [**] or p<0.001 [***], two-tailed t-test).

Some reports of cotranslational integration of membrane proteins into liposomes demonstrate a liposome-dependent increase in the total amount of protein synthesized [7], [34] whereas others detect no such liposome enhancement of translation rate [8]. We addressed this issue by conducting cell-free translation reactions of [^35^S]AAC in the presence of increasing liposome concentrations and measuring the total amount of protein synthesis. We found a marked increase in total [^35^S]AAC synthesis with increasing concentrations of inner membrane-mimetic liposomes (containing CL) but interestingly this stimulating effect was absent for liposomes lacking CL (Fig. 4A, top panel). As a control, we measured the rate of translation of the soluble protein Tim9 under identical conditions and found no liposome-dependent enhancement on protein synthesis (Fig. 4A, bottom panel). We therefore conclude that the presence of CL-containing liposomes, but not liposomes lacking CL, stimulates the translation rate of AAC in our *in vitro* translation system.

To ascertain whether the presence of CL in liposomes also enhanced the membrane integration of AAC, we conducted AAC translation reactions in the presence of SUVs containing varying molar amounts of CL and purified them by SGU. The amount of [^35^S]AAC associated with liposomes containing small amounts of CL was relatively low, but increased with a threshold-type response between 15 and 20 mol% CL and plateaued at higher values (Fig. 4B). To determine whether this CL-dependent enhancement of AAC association reflected *bona fide* integration, we prepared [^35^S]AAC-proteoliposomes containing 0, 16, and 24 mol% CL and measured the liposome-associated (SGU-purified) fractions, as well as the carbonate- or urea-resistant fractions recovered with the membrane. Consistent with the above results (Fig. 4B), the amount of [^35^S]AAC that floated with the liposomes increased in the presence of CL, but saturated at higher values (Fig. 4C, left panel). Further, the membrane-associated fractions of AAC that were carbonate- and urea-resistant (Fig. 4C, right panel, white and hatched bars, respectively) showed the same CL-dependent trend, indicating that the CL content of liposomes enhanced the amount of fully integrated AAC. We therefore conclude that the presence of CL in the liposomes included in our *in vitro* translation system increases both the rate of translation and the efficiency of integration of AAC.

### III. The integration of AAC into liposomes occurs cotranslationally

**Figure 5:**
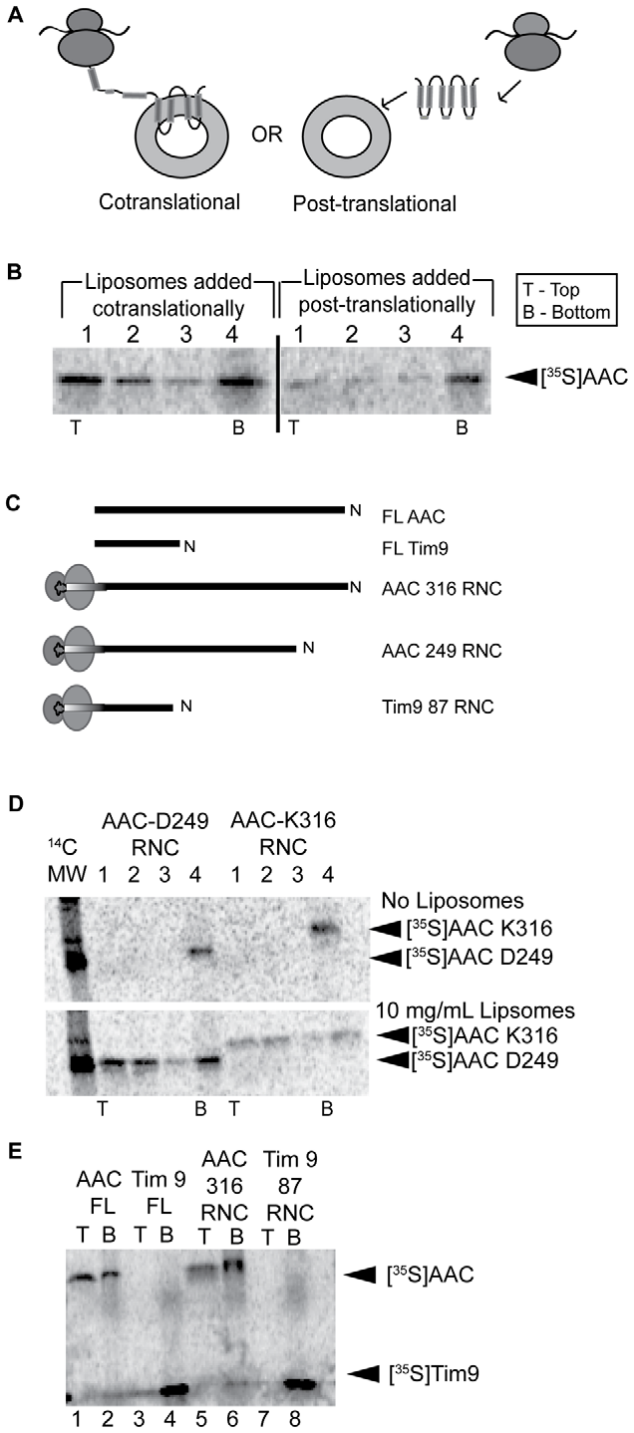
Liposome-assisted integration of AAC occurs cotranslationally. A) Schematic representation of cotranslational and post-translational modes of membrane protein integration into liposomes. B) Liposomes (final concentration of 10 mg/mL) were added to [^35^S]AAC translation reactions during translation or after the termination of protein synthesis as indicated, reactions were subjected to SGU, and fractions (1–4 from top to bottom, as indicated) were resolved by SDS-PAGE as in Fig. 1. C) Schematic of full length and ribosome-bound nascent chains analyzed in panels D and E. “AAC 249 RNC” and “AAC 316 RNC” intermediates have ribosome nascent chain lengths of 249 and 316 amino acids, respectively. “Tim9 87 RNC” has a chain length of 87 amino acids and is truncated just before the native stop codon. D) Ribosome-bound nascent chain constructs of AAC ([^35^S]AAC 249 and 316 intermediates) were translated in the absence (top panel) or presence (bottom panel) of liposomes and subjected to SGU fractionation as in panel B. E) Full length and ribosome-bound intermediates of AAC and Tim9 were translated in the presence of liposomes and subjected to SGU, and the top and bottom fractions of the gradient were resolved by SDS-PAGE.

The spontaneous integration of *in vitro* translated polypeptides into liposomes could in principle occur either cotranslationally (i.e., integration occurs while the polypeptide is being synthesized on the ribosome) or post-translationally (i.e., integration occurs after polypeptide release from the ribosome) (Fig. 5A). Previous reports have concluded that such unassisted integration occurs cotranslationally because when liposomes were added after cell-free translation ended, the synthesized proteins did not co-isolate with the liposomes [7], [8], [35]. To address this issue in our system, [^35^S]AAC was translated in the presence of liposomes or it was translated in the absence of liposomes, treated with cyclohexamide to halt translation and then incubated with liposomes. SDS-PAGE analysis of the resulting sucrose density gradient fractions confirmed that liposomes must be present while translation is occurring in order for [^35^S]AAC to float with the vesicles (Fig. 5B), consistent with previous results (i.e., Fig. 2 of reference [8]). We note also that the liposome-dependent enhancement of AAC translation observed above (Fig. 4A) is evident here by an increase in the total synthesized AAC when liposomes are included in translation (Fig. 5B, left) relative to when liposomes are added after translation termination (Fig. 5B, right).

To independently test for the ability of AAC to interact with liposomes during translation (i.e., while still bound to the ribosome) we prepared ribosome-bound nascent chain (RNC) intermediates by programming translation with mRNA transcripts truncated within the coding region. Because these transcripts lack a stop codon, normal translation termination does not occur and nascent polypeptides remain bound to ribosomes by stable peptidyl tRNA ester bonds [36]–[38] (Fig. 5C). Because the ribosome tunnel accommodates ∼30–40 amino acids of the elongating chain (assuming a fully extended conformation) [39]–[41], nascent chains longer than this will expose N-terminal residues to the medium. [^35^S]AAC RNC intermediates either 249 or 316 residues in length that were translated in the absence of liposomes were recovered in the pellet fraction after SGU (Fig. 5D, upper panel); however, when translated with liposomes, these RNCs co-isolated with liposomes at the top of the gradient (Fig. 5D, bottom panel), indicating a strong membrane interaction even for ribosome-bound polypeptides. To confirm that this liposome interaction was not due to nonspecific binding of ribosomes to the vesicles, the same assay was conducted with RNCs of the soluble protein Tim9 (Fig. 5E). Whereas RNC truncates of AAC associated as strongly with liposomes as full-length AAC (Fig. 5E, compare lanes 5 and 6 with lanes 1 and 2), Tim9 did not float with liposomes either as a ribosome-bound truncate or as released polypeptide (Fig. 5E, compare lanes 7 and 8 with lanes 3 and 4). We therefore conclude that the hydrophobic polypeptide, not the ribosome, mediated the strong liposome interactions for AAC constructs and, more generally, that spontaneous membrane interaction in the cell-free system occurs cotranslationally.

## Discussion

Cell-free expression systems are emerging as powerful approaches to the production of membrane proteins and characterization of their structure and function [1], [2], [42]–[44]. In the past few years, several groups have independently shown that *in vitro* translated membrane proteins can directly integrate into model membrane systems including synthetic liposomes [7], [9], [11], [34], [45]–[48], and nanoscale lipid bilayers (nanodiscs) [49], [50] that are included in the synthesis reaction in an manner that does not require detergents. In this report, we have shown that the AAC transporter also integrates spontaneously into SUVs in a cell-free translation, which, to the best of our knowledge, represents the first such study for a mitochondrial inner membrane protein.

We demonstrated the liposome association and integration of *in vitro* translated AAC using a host of established methods. First, nearly 50% of [^35^S]AAC synthesized in the presence of liposomes floated at the top of a sucrose density gradient (Fig. 1), confirming the association of the protein with buoyant vesicles. Second, we found that approximately half of the [^35^S]AAC that were co-isolated with liposomes were resistant to carbonate extraction (Fig. 2A), a primary diagnostic indicator of stably integrated membrane proteins. Finally, we used a cysteine accessibility approach with the thiol-reactive fluorescent probe IANBD to show that a cysteine residue in an AAC transmembrane segment is specifically shielded from labeling when integrated into liposomes, whereas a cysteine site in a soluble loop region is labeled to a similar extent regardless of liposome association (Fig. 2B). Taken together, these data suggest that a substantial amount of AAC was integrated into liposomes during cell-free translation. However, because only about half of our translated AAC was resistant to carbonate extraction, a significant portion of AAC must have remained nonspecifically bound to liposomes even after SGU. Hence, one should exercise caution when attempting to use this method to generate homogeneous populations of integrated membrane proteins. With that caveat, it is clear that our wheat germ based *in vitro* translation system is capable of supporting the integration of AAC into synthetic liposomes.

The most critical test for the proper folding and integration of reconstituted membrane proteins is functionality; therefore, it is important that we confirmed the transport activity of AAC that was translated in our system (Fig. 3). AAC in isolated mitochondria and reconstituted into proteoliposomes has been amply shown to mediate the 1∶1 electrogenic exchange of ATP^4−^ and ADP^3−^ [51]–[54]. The assay used in the present work measured the AAC-dependent transport of ATP out of vesicles, which was likely driven in part by the external ADP present in the translation reaction. Although this system in its current form does not allow for the precise measurement of ADP/ATP exchange parameters, it did unequivocally demonstrate functionally active carriers in our proteoliposomes, because: (i) ATP efflux was blocked by the specific AAC inhibitor CAT (Fig. 3B), and (ii) ATP efflux was stimulated by an added incubation with external ADP (Fig. 3C).

Nearly all membrane proteins synthesized within the physiological context of the cell require some combination of soluble chaperones and membrane-bound complexes to mediate their integration into the lipid phase. Like other metabolite carrier proteins of the inner mitochondrial membrane, AAC is encoded in nuclear DNA, synthesized on cytosolic ribosomes, and subsequently targeted to the mitochondria [16], [55]. Carrier proteins are directed to mitochondrial import complexes by multiple internal targeting sequences that generally exist within the segments that ultimately form their six transmembrane helices [56]. At the organelle surface, AAC engages the TOM (Translocase of the Outer Mitochondrial membrane) complex, which mediates its translocation across the outer membrane. Within the aqueous intermembrane space, AAC is kept in a soluble state by association with the hexameric Tim9-Tim10 chaperones [16], [17]. Finally, AAC is directed to the TIM22 complex, where it integrates into the IM *via* TIM22 channels in a manner dependent upon the membrane potential [57].

Given the complexity of *in vivo* biogenesis pathways such as this, it is perhaps surprising that membrane proteins synthesized in a cell-free system [7], [9], [11], [34], [45]–[48], AAC now included, could integrate into synthetic liposomes in the absence of dedicated translocation/integration complexes. Yet this may be explained by several characteristics of the cell-free reactions. (i) The presence of soluble chaperones in cell-free systems may maintain synthesized proteins in an integration-competent conformation. For example AAC has been shown to require association with Hsp70 and Hsp90 chaperones in the cytosol to maintain solubility and facilitate targeting [58], [59]. Cell-free translations, such as the wheat germ lysate-based system used in the present study, are rich in chaperones. In fact, in their study on the insertion of stearoyl-CoA desaturase into liposomes, Fox and colleagues showed that Hsp70 from their wheat germ translation system strongly associated with their density gradient purified proteoliposomes [9]. (ii) Native biomembranes contain a complex mix of lipids, some of which may inhibit the unassisted integration found in synthetic liposomes. For example, many signal-anchored and tail-anchored proteins targeted to the mitochondrial outer membrane appear to integrate in the absence of translocation complexes [60]; however, the presence of ergosterol strongly inhibits their insertion, likely owing to the increased rigidity and reduced compressibility that this sterol imparts to the bilayer [61], [62]. As another example, the otherwise spontaneous integration of M13 procoat and Pf3 coat proteins into liposomes is blocked by diacylglycerol, which, due to its bulky structure, was proposed to occupy conical crevices where acyl chains are exposed to the bilayer surface due to repulsion among phospholipid head-groups [35], [63]. (iii) Biological membranes are very protein rich; in the case of the mitochondrial IM containing a protein-to-phospholipid ratio up to 4∶1 by weight [64]. This feature may limit the access of newly synthesized proteins to the lipid bilayer, whereas synthetic liposomes may provide a more accessible surface for direct lipid contact. (iv) Unassisted protein integration during cell-free synthesis is obligatorily cotranslational ([7], [8] and Fig. 5 of this study). *In vivo*, the bilayer insertion of many membrane proteins, including AAC, occurs after polypeptide release from the ribosome, requiring specialized chaperones to prevent aggregation. In contrast, the vectorial presentation of nascent chains directly to the bilayer (i.e., one TMS at a time, as could occur in a cotranslational mode) may facilitate integration. In this regard, the staging of nascent chain folding could also be important. The ribosome tunnel proves an environment for the folding of nascent chain regions with high α-helical propensity [65]–[67] and hydrophobic TMSs have been shown to fold into compact structures within the ribosome [68], [69]. Upon exit from the ribosome, TMSs have been shown to lose their compact secondary structure, but not when in the presence of a membrane-bound translocon [68]. Consistent with computational predictions [70], this indicates that the ribosome tunnel is important in nucleating and stabilizing secondary structure of hydrophobic TMSs. We suggest that in the present study, synthetic liposomes present during translation may have provided a hydrophobic environment to stabilize the α-helical structures of the TMSs as they emerged from the ribosome tunnel.

The lipids that reside within the bilayer are, of course, critical determinants of membrane protein folding and topology. The role of a lipid as a molecular chaperone is best illustrated by the work of Dowhan and colleagues, who have demonstrated that the zwitterionic nonbilayer lipid phosphatidylethanolamine is critical in reversibly promoting the folding and topogenesis of lactose permease [71]–[74]. Other studies have shown the importance of lipids in mediating polypeptide integration. In their study on the spontaneous integration of the Pf3 procoat protein, de Kruijff and coworkers found that CL, in a concentration-dependent manner, stimulated the efficiency of insertion, although the effect was attributed to the anionic character of the lipid, not its shape [75]. More recently it was shown that the spontaneous integration of the polytopic protein bacteriorhodopsin into liposomes during cell-free synthesis required lipids with a specific acyl chain length for optimal insertion [7].

Interestingly, in our system, the extent of AAC integration was strongly dependent upon the presence of CL, with a threshold concentration corresponding to the 15–20 mol% that is consistent with the CL concentration that exists naturally in the mitochondrial IM [23], [25]. CL possesses several physiochemical characteristics that may account for this effect. In the presence of divalent cations, CL is a non-bilayer lipid with propensity for inverted hexagonal (H_II_) phase [76]. This corresponds to a molecular geometry with a small effective size of the headgroup relative to the volume occupied by the acyl chains. When such non-bilayer forming lipids exist within the context of a lamellar bilayer (e.g., liposomes), this creates tension within the planar membrane that may expose the hydrophobic core to the aqueous phase, which may have promoted the polypeptide integration that we observe in our translation system.

Finally, we note that the presence of CL in the liposomes included in our cell-free translations not only stimulated the rate of AAC integration, but also enhanced the overall polypeptide synthesis rate as well. We observed that CL-containing vesicles increased the production of hydrophobic protein (AAC, Fig. 4A, upper panel) but not soluble protein (Tim9, Fig. 4A lower panel). Given our observation that AAC initiates integration in our system while still ribosome bound (Fig. 5), it appears that the presence of CL-containing liposomes in the translation somehow (possibly by chaperone-like activity) enhanced the efficiency with which ribosomes translated the hydrophobic AAC polypeptide. We suggest that by providing a platform for stable integration, CL-containing liposomes prevented nascent chain misfolding or aggregation that may have been inhibitory to the translation apparatus.

In this report, we have shown that a multispanning protein of the inner mitochondrial membrane translated in a cell-free system is capable of integration into synthetic unilamellar vesicles. We have demonstrated that both the translation rate and integration efficiency of *in vitro* translated AAC are dependent on the presence of CL in the liposomes, suggesting a possible chaperone-like role for this lipid, as has been suggested for PE. Finally, we provide strong evidence that integration in this cell-free system occurs cotranslationally. The reductionist system used in the present work does not provide insights into the details of the *in vivo* biogenesis pathway of mitochondrial inner membrane proteins, which requires the TIM22 protein translocase and an energized IM. However, this cell-free system does provide an excellent means of synthesizing proteoliposomes by a detergent-free process for structural and functional studies of membrane proteins within a lamellar bilayer.

## Materials and Methods

### Plasmid preparation

Sequences encoding the polypeptides used in this study were PCR-amplified from *S. cerevisiae* genomic DNA and subcloned into pSP65 (Tim9 and Tim12) or pGEM4Z (AAC) vectors. Variants of AAC were prepared using QuickChange site-directed mutagenesis (Stratagene): AAC ΔCys was created by substituting all native Cys residues with Ala and monocysteine mutants were created by inserting in-frame Cys codons at the selected site within AAC ΔCys background.

### Preparation of liposomes

Our procedure for liposome formation was derived from several published sources [8], [9], [61], [77]. Synthetic phospholipids were purchased as chloroform stocks from Avanti Polar Lipids (Alabaster, AL). The following lipids in the indicated molar ratios were prepared as a biomimetic of the mitochondrial IM: 1-palmitoyl-2-oleoyl-*sn*-glycero-3-phosphocholine (POPC, 54%), 1-palmitoyl-2-oleoyl-*sn*-glycero-3-phosphoethanolamine (POPE, 24%), 1′,3′-bis[1,2-dioleoyl-*sn*-glycero-3-phospho]-*sn*-glycerol (CL, 16%), 1-palmitoyl-2-oleoyl-*sn*-glycero-3-phosphoserine (POPS, 4%), and 1-palmitoyl-2-oleoyl-*sn*-glycero-3-phosphate (POPA, 2%). For blends containing different molar amounts of CL, the amount of POPC was adjusted accordingly. Lipid mixtures were dried under a nitrogen stream for 15–30 min and then evaporated overnight in a vacuum desiccator to remove all organic solvent. The lipid film was rehydrated in hydration buffer (10 mM Tris-HCl, pH 7.4, 100 mM NaCl, 2 mM MgCl_2_) [77] for 30 min at room temperature with vortexing to resuspend the lipid film to a final concentration of 25 mg/ml lipid. The suspension was then passed 17 times through a Mini-Extruder (Avanti) with a 0.1 micron track-etch polycarbonate membrane (Nucleopore, Pleasanton, CA) to create uniformly sized small unilamellar vesicles.

### Cell-free Transcription

mRNA was transcribed as described [78]–[80] from PCR-generated DNA fragments using 5′ and 3′ oligonucleotides complementary to the plasmid SP6 promoter and Tim23/pSu9DHFR coding sequences. PCR products were transcribed *in vitro* with SP6 polymerase at 37°C for 1.5 hr in reactions containing 100 mM HEPES-KOH (pH 7.5), 20 mM MgCl_2_, 2.5 mM spermidine, 12 mM dithiothreitol (DTT), 4 mM each of ATP, CTP, and UTP, 0.4 mM GTP, 0.013 U/µL G(5′)ppp(5′)G RNA cap analog (New England Biolabs), 0.5 U/µL RNasin, and 0.006 U/µL pyrophosphatase, then supplemented with 4 mM GTP and allowed to proceed an additional 0.5 hr. mRNA was precipitated overnight at −20°C in ethanol and 90 mM sodium acetate (pH 5.2), washed in 70% (v/v) ethanol, and reconstituted in TE buffer (10 mM Tris-HCl, 1 mM EDTA, pH 7.5).

### Cell-free Translation

Translation reactions were conducted using a wheat germ lysate prepared in-house by an established protocol [81]. Translation reactions (total volume 100 µL) were programmed with AAC, Tim9 or Tim12 mRNA transcripts [8%(v/v)] in a reaction including 20 mM HEPES-KOH (pH 7.5), 100 mM potassium acetate (pH 7.5), 1.5 to 2.5 mM magnesium acetate, 1 mM DTT, 200 µM spermidine, 8 µM S-adenosylmethionine, protease inhibitors [0.25 µg/mL each of leupeptin, chymostatin, antipain and pepstatin A and 0.025%(v/v) aprotinin], 0.2 U/µL RNAsin, 1.2 mM ATP, 1.2 mM GTP, 64 mM creatine phosphate, 9.6 U/nL creatine phosphokinase, 30 µM of amino acids (but lacking methionine to enhance incorporation of [^35^S]methionine), and 20% (v/v) wheat germ extract. Translation reactions were incubated at 26°C for 5 min prior to the addition of mRNA, 0.13 µCi/µL [^35^S]methionine and 10 mg/mL SUVs (unless otherwise noted) and then continued at 26°C for 40 min as described [78], [82].

### Proteoliposome purification and extraction procedures

SGU was used to isolate proteoliposomes from the translation mixture and unincorporated protein as described [8], [83]. Following translation, samples were placed on top of a discontinuous sucrose gradient (5–30% sucrose steps depending on the experiment) or sucrose cushion (15% sucrose) in 11×34 mm polyallomer tubes, suspended in corresponding buckets in a TLS 55 swinging bucket rotor (Beckman Coulter) and centrifuged at 163,000× *g* (55,000 rpm) for 2 hr at 15°C. To separate peripherally associated proteins from stably integrated ones, SGU-purified proteoliposomes were subjected to extraction procedures with carbonate or urea as described [5], [84], [85]. Samples were diluted 1∶10 in 0.1 M sodium carbonate (pH 11.5) or 1∶7 in 4.5 M urea, incubated on ice for 30 min and centrifuged at 150,000× *g* for 30 min. Pellets were resuspended in SDS-PAGE sample buffer and supernatants were TCA precipitated as described [5].

### Thiol labeling

Translation reactions or SGU-purified proteoliposomes containing AAC ΔCys or monocysteine variants were added to reactions in buffer (10 mM Tris-HCl, pH 7.4, 100 mM NaCl, 2 mM MgCl_2_) containing 75 µM IANBD [*N*,*N′*-dimethyl-*N*-(iodoacetyl)-*N*′-(7-nitrobenz-2-oxa-1,3-diazol-4-yl) ethylenediamine] (Molecular Probes, Eugene, OR) and incubated at room temperature in the dark for variable lengths of time. To terminate labeling, reactions were quenched by addition of 0.2 M DTT.

### SDS-PAGE and gel analysis

Samples were diluted in an equal volume of SDS-PAGE sample buffer (140 mM Trizma base, 20% [v/v] glycerol, 4% [w/v] sodium dodecyl sulfate, 0.05% [w/v] bromophenol blue, 0.25 M DTT) and resolved on 12.5% SDS-PAGE gels (except for IANBD-labeled samples, which were run on 15% gels). All imaging was performed using a Pharos FX Plus Molecular Imager (BioRad). Radiolabeled samples were visualized using a phosphor Imaging Screen-K (Kodak) and NBD-labeled samples were visualized by in-gel fluorescence (excitation laser 488 nm, 530 nm emission filter). Image analysis and quantitation was done using Quantity One software.

### Luciferin-Luciferase Luminescence Assay

SUVs were prepared as above, but lipid films were rehydrated in hydration buffer containing 20 mM ATP (pH 7.4). The resulting ATP-loaded liposomes were added to AAC translation reactions. Where indicated, 10 µM CAT (Sigma) was added to the translations. Proteoliposomes were then subjected to gel filtration chromatography (Sephadex G50) to remove unencapsulated ATP. Where indicated, column purified proteoliposomes were treated with 200 µM ADP and incubated for 30 min at 26°C followed by a second column purification. Vesicles were treated with 0.2% triton X-100 (to disrupt the vesicles) or buffer only (to leave vesicles intact), and the amount of free ATP was quantified using a commercially available kit (ATP Determination Kit, Molecular Probes) following the manufacturer's instructions and the resulting luminescence was read at 560 nm. Based on ATP calibration curves, luminescence readings from all samples were in the linear response range.
